# The Number of MRGPRX2-Expressing Cells Is Increased in Skin Lesions of Patients With Indolent Systemic Mastocytosis, But Is Not Linked to Symptom Severity

**DOI:** 10.3389/fimmu.2022.930945

**Published:** 2022-07-26

**Authors:** Polina Pyatilova, Tameem Ashry, Yanyan Luo, Jiajun He, Hanna Bonnekoh, Qingqing Jiao, Sherezade Moñino-Romero, Man Hu, Jörg Scheffel, Stefan Frischbutter, Maud A. W. Hermans, Bradford A. Youngblood, Marcus Maurer, Frank Siebenhaar, Pavel Kolkhir

**Affiliations:** ^1^ Institute of Allergology, Charité – Universitätsmedizin Berlin, Corporate Member of Freie Universität Berlin, Humboldt-Universität zu Berlin, and Berlin Institute of Health, Berlin, Germany; ^2^ Allergology and Immunology, Fraunhofer Institute for Translational Medicine and Pharmacology ITMP, Berlin, Germany; ^3^ Department of Pathology, College of Medicine, King Saud University, Riyadh, Saudi Arabia; ^4^ Department of Dermatology, The Affiliated Hospital of Southwest Medical University, Luzhou, China; ^5^ Department of Dermatology, The First Affiliated Hospital of Soochow University, Suzhou, China; ^6^ Department of Internal Medicine, Section Allergy and Clinical Immunology, Erasmus University Medical Center, Rotterdam, Netherlands; ^7^ Allakos Inc., San Carlos, CA, United States

**Keywords:** MRGPRX2, cortistatin, mastocytosis, mast cell, mRNA, major basic protein (MBP), skin, symptoms

## Abstract

**Background:**

Recently, the expression of the mast cell (MC) receptor Mas-related G protein–coupled receptor X2 (MRGPRX2) has been detected in lesional skin of adult patients with cutaneous mastocytosis. As of yet, little is known about the clinical relevance of MRGPRX2 and its agonists in patients with mastocytosis, including indolent systemic mastocytosis (ISM).

**Methods:**

MRGPRX2 and MRGPRX2 agonists, cortistatin (CST), and major basic protein (MBP) were analyzed in lesional and non-lesional skin of patients with ISM and skin of healthy controls by immunohistochemistry. Co-localization of MRGPRX2 and MRGPRX2-mRNA with the MC marker tryptase was assessed by immunofluorescence microscopy and *in situ* hybridization, respectively. We assessed clinical, demographic, and laboratory data, including mastocytosis activity score (MAS), serum tryptase, and *KIT* D816V allele burden.

**Results:**

The number of MRGPRX2-expressing (MRGPRX2+) cells, MRGPRX2-mRNA+ MCs, and CST-expressing (CST+) and MBP-expressing (MBP+) cells was significantly higher in lesional skin as compared to non-lesional skin and/or skin of healthy controls (all p < 0.05). Increased numbers of MRGPRX2+ cells, MRGPRX2-mRNA+ MCs, and CST+ and MBP+ cells were not associated with clinical and laboratory features of ISM, including disease burden, symptom severity, evidence of anaphylaxis, and tryptase levels.

**Conclusions:**

Skin lesions of patients with ISM showed high numbers of MRGPRX2+ cells, although they were not linked to symptom severity. Clinical relevance of the MRGPRX2-mediated pathway of MC activation in ISM remains unclear and should be investigated in further studies.

## Introduction

Systemic mastocytosis (SM) is a rare disease characterized by infiltration of clonally derived mast cells (MCs) in bone marrow, skin, the gastrointestinal tract, and other organs. The prevalence of SM in the general population varies from 10 to 17 cases per 100,000 people (1–3). Indolent systemic mastocytosis (ISM) is the most common form of SM found in up to 91% of adult patients and is associated with a favorable prognosis (2, 4, 5). ISM is diagnosed when spleen and liver impairment is absent and no more than one B-finding is present, i.e., >30% MC infiltration in the bone marrow and serum tryptase level >200 ng/ml—signs of dysmyelopoiesis without substantial cytopenias and/or organomegaly (5). Skin involvement has been reported in about 95% of ISM patients, often associated with symptoms including itching, whealing, and flushing (6, 7). Severe anaphylaxis triggered by hymenoptera venom, food, or drug components is observed in half of ISM patients (2).

ISM symptoms can induce a significant impairment of patients’ quality of life (4, 8). Avoidance of known symptom triggers and antimediator therapies are not always effective in ISM patients, whereas cytoreductive therapies and non-selective tyrosine kinase inhibitors can be associated with severe adverse effects (9). Although there are some novel drugs in development for ISM, no curative treatment for the disease exists (10). Thus, there is a high unmet need to identify markers associated with disease severity and poor response to treatment and investigate new MC-associated targets for development of effective and safe therapies.

MCs, primary effector cells in mastocytosis, express a wide range of receptors and release a variety of mediators upon activation (10). The best explored pathway of MC activation is linked to allergen-induced IgE crosslinking of the high-affinity FcϵRI receptor, which drives IgE-mediated anaphylactic reactions associated with mastocytosis (11). Also, IgE-independent MC activation could lead to clinically relevant reactions, particularly associated with drug intolerance, *via* the Mas-related G protein–coupled receptor X2 (MRGPRX2) on MCs (12). Increased MRGPRX2 expression on skin MCs has been recently reported in patients with maculopapular cutaneous mastocytosis and suggested as a possible driver of mastocytosis pathogenesis (13).

MRGPRX2 is a multiligand receptor expressed by MCs (14), which responds to a broad variety of exogenous and endogenous stimuli, e.g., insect venom, drugs, neuropeptides such as cortistatin (CST) and substance P (SP), eosinophil granule proteins including major basic protein (MBP) and eosinophil cationic protein (ECP), and infections (15–19). Insect venom and quinolone antibiotics are known triggers of anaphylaxis in ISM patients. Mastoparan, an insect venom constituent, and quinolones are also MRGPRX2 agonists that might point toward a contribution of MRGPRX2-mediated MC activation to anaphylaxis development in some ISM patients (14, 20). For example, the MRGPRX2-mediated pathway was hypothesized in a patient with ISM who developed anaphylaxis to ciprofloxacin (21). On the other hand, FcϵRI but not MRGPRX2 was overexpressed on bone marrow MCs in patients with clonal MC disorders, mostly ISM, and wasp venom anaphylaxis, compared to those without anaphylaxis (22). Therefore, the role and relevance of MRGPRX2-mediated MC activation and MRGPRX2 agonists in ISM are still unclear and require further investigation.

Here, we assessed whether MRGPRX2 and its agonists, namely, CST and MBP, are expressed in the skin of ISM patients and whether their expression in skin is linked to clinical features and laboratory markers of ISM.

## Materials and methods

### Study Population

Skin samples (lesional n = 22, non-lesional n = 21) from 22 adult patients with ISM, who participated in a clinical trial (ClinicalTrials.gov identifier: NCT02808793) were included in a *post hoc* analysis to assess expression of MRGPRX2 and MRGPRX2 agonists. All patients were diagnosed in accordance with the 2016 WHO criteria (5). Skin samples of 10 healthy controls (HCs) were included from the biobank of Charité – Universitätsmedizin Berlin (ethics approval EA1/407/16) and further analyzed for CST (n = 10) and MRGPRX2 (n = 8) expression in the skin. All participants signed an informed consent.

Baseline serum tryptase levels (ImmunoCAP) and *KIT* D816V allele burden in blood (allele-specific quantitative polymerase chain reaction/ASO-qPCR) were assessed on a regular basis in Labor Berlin-Charité/Vivantes. Symptoms, i.e., itching, whealing, flushing, diarrhea, abdominal pain, musculoskeletal pain, fatigue, headache, and difficulty concentrating, as well as total disease activity were assessed using the Mastocytosis Activity Score (MAS) (6). The Questionnaire was completed daily by the patients for at least 1 week. Afterward, total scores and domain scores were calculated. MAS total score ranges <19, 19–34, and >34 were counted as mild, moderate, and severe disease severity, respectively, as described before (6).

The extent of skin involvement was analyzed independently by two experienced physicians. The mastocytosis in the skin (MIS) scoring system was developed. Patients were divided into five groups, due to the number and color intensity of lesions in the most involved region, where 0 is no skin lesions and 5 is very severe skin involvement (**Supplementary Figure 1**). The evidence of anaphylaxis and osteopenia/osteoporosis was retrospectively extracted from the medical files. Patients were under standard dosed H1-antihistamine therapy, but no topical treatment or ultraviolet irradiation was applied.

### Immunohistochemistry and Immunofluorescence

CST and MRGPRX2 staining was performed, as described earlier (23). Briefly, after embedding in paraffin *via* standard protocol, 5-μm sections were baked at 60°C for 1 h in an incubator. Deparaffinization and rehydration were performed using regular procedures. Heat-induced antigen retrieval citrate buffer was applied for the staining with anti-CST (sc-393108, A-7; Santa Cruz Biotechnology, Dallas, TX, USA). Enzymatic pretreatment was used for anti-MRGPRX2 (ab167125, Abcam, Cambridge, MA, USA) staining. Further procedures were performed according to the manufacturer’s instructions (Dako EnVision+, Peroxidase). Washing steps in between were performed. Finally, slides were stained with Mayer’s hematoxylin (ab128990, Abcam). Lack of the primary antibody was used as a negative control.

MBP expression was assessed in skin of ISM patients only. For MBP staining, enzymatically pretreated paraffin sections were incubated with anti-MBP (Bio-Rad, Hercules, CA, USA, MCA5751) at 4°C overnight. Alkaline phosphatase IgG polymer (MP-5402, Vector, Burlingame, CA, USA), detected with Vector Red Substrate Kit (VEC-SK-5100, Biozol, Eching, Germany), was used as a secondary antibody. Other steps were performed as described above. MCs were identified with the use of anti-tryptase (M7052; Dako, Glostrup, Denmark) according to the manufacturer’s instructions.

Co-localization of MRGPRX2 with the MC marker tryptase was assessed by immunofluorescence. The sections were prepared, as described above with the use of heat-induced antigen retrieval and incubated with a mixture of anti-MRGPRX2 (LS-A6644; LSBio, Seattle, WA, USA) and anti-tryptase (M7052; Dako) at 4°C overnight. The next day, slides were incubated in a mixture of two fluorescent conjugated secondary antibodies, Alexa Flour 488-conjugated goat anti-mouse and Rhodamine (TRITC)-conjugated goat anti-rabbit, diluted in TBS containing 2% goat normal serum, for 30 min in the dark at room temperature. In between slides were washed three times in TBS. Mounting with preserve reagent including DAPI (00-4959-52; Invitrogen) was applied afterward.

### *In Situ* Hybridization

*In situ* hybridization (ISH) of MRGPRX2-mRNA was conducted in line with RNAscope Single-Plex Red Chromogen V2.5 protocol and the recommended HybEZ oven and in combination with materials for immunohistochemistry (IHC)/immunofluorescence (IF). ISH was completed prior to any IHC/IF protocol. All tissue was prepared in line with RNAscope FFPE protocol with antigen retrieval being performed for skin at 65°C for 20.5 h up to 21 h maximum in an oven. The remainder of the RNAscope protocol was followed as per the ACDBio manuals (Documents 322452 and 322360-USM). RNAscope negative and positive controls were used to ensure the quality of yield in this approach. IHC/IF tryptase co-staining was conducted after ISH red chromogen development at the step for protein block. Red chromogen was visible using a fluorescence microscope (BZ-X800; Keyence, Itasca, USA), and positive and negative controls were used to calibrate fluorescence excitation to represent the chromogen stain. After adjusting the settings, they were then used for imaging of the entire assay.

### Quantitative Histomorphometry

All sections were blinded and assessed independently by two investigators. The number of positive cells was manually counted in at least five horizontally adjacent high-power microscopic fields (HPF) in the upper papillary dermis. Mean values “per HPF” were calculated and further converted to “per mm^2^” (CST^+^ cells ×200, 0.6 mm^2^, MRGPRX2^+^ cells ×200, 0.42 mm^2^, MCs ×400, 0.2 mm^2^, and MRGPRX2-mRNA^+^ MCs ×400, 0.1 mm^2^).

### Statistical Analysis

We used the Statistical Package for the Social Science (IBM SPSS version 25.0; IBM Corp, New York, NY), R (R Foundation for Statistical Computing), and GraphPad Prism 8 (GraphPad Software, La Jolla, CA). Demographic characteristics of patients with ISM were reported using descriptive statistics. Data distribution was analyzed with the Kolmogorov–Smirnov test. Normally and not normally distributed data were presented as mean ± SD and median (interquartile range), respectively. The Spearman correlation coefficient was applied to assess correlations. Statistically significant differences between groups were calculated using t-test for normally distributed data and the Mann–Whitney U test for non-normally distributed data. Differences between lesional and non-lesional parameters were analyzed with the paired t-test (normally distributed data) or the Wilcoxon signed-rank test (not normally distributed data). For all tests, P < 0.05 was considered as statistically significant.

## Results

### Demographic, Clinical, and Laboratory Characteristics

We studied 22 patients with ISM (72.7% women) and 10 HCs (40.0% women) with mean age of 50.6 ± 9.3 and 51.6 ± 14.2 years, respectively. Skin lesions, i.e., MIS, were present in all ISM patients. Osteopenia/osteoporosis and anaphylaxis were common signs associated with mastocytosis, present in 70.0% and 42.9% of patients, respectively (**Table 1**). The median baseline serum tryptase level (bST) and *KIT* D816V allele burden were 31.0 (IQR: 21.0–109.5) ng/ml and 1.0 (0.3–2.6)% in the blood of ISM patients, respectively. Disease activity was mild (27.3%, n = 6), moderate (31.8%, n = 7), and severe (40.9%, n = 9), based on the MAS total values. Detailed demographic and clinical characteristics of ISM patients and HCs are presented in **Supplementary Tables 1, 2**.

**Table 1:** Demographic, clinical, and laboratory characteristics of ISM patients and healthy controls.

Parameter	ISM, n = 22	HCs, n = 10
**Age**, years, mean ± SD (range)	50.6 ± 9.3 (33.0–65.0)	51.6 ± 14.2 (23.0–68.0)
**Gender**, female, n/total (%)	16/22 (72.7)	4/10 (40.0)
**Anaphylaxis**, n/total (%)	9/21 (42.9)	–
**Osteoporosis/osteopenia**, n/total (%)	14/20 (70.0)	–
**Skin lesions (i.e., MIS)**, n/total (%)	22/22 (100.0)	–
**MAS total,** mean ± SD (range)	31.4 ± 14.6 (9.0–54.0)	–
**Serum baseline tryptase, ng/ml,** median (IQR)	31.0 (21.0–109.5)	5.2 (4.2–6.3)
***KIT* D816V in PB, yes,** n/total (%)	21/22 (95.5)	–
***KIT* D816V burden in PB, %,** median (IQR)	1.0 (0.3–2.6)	–

ISM, indolent systemic mastocytosis; HCs, healthy controls; MIS, mastocytosis in the skin; MAS, mastocytosis activity score; PB, peripheral blood; –, not applicable/no information.

### The Number of MRGPRX2-, CST-, and MBP-expressing Cells is Increased in Lesional Skin of Patients With Indolent Systemic Mastocytosis

The numbers of MRGPRX2+, CST+, and MBP+ cells were assessed in lesional and non-lesional skin of ISM patients and skin of healthy controls by immunohistochemistry.

**Figure 1:**
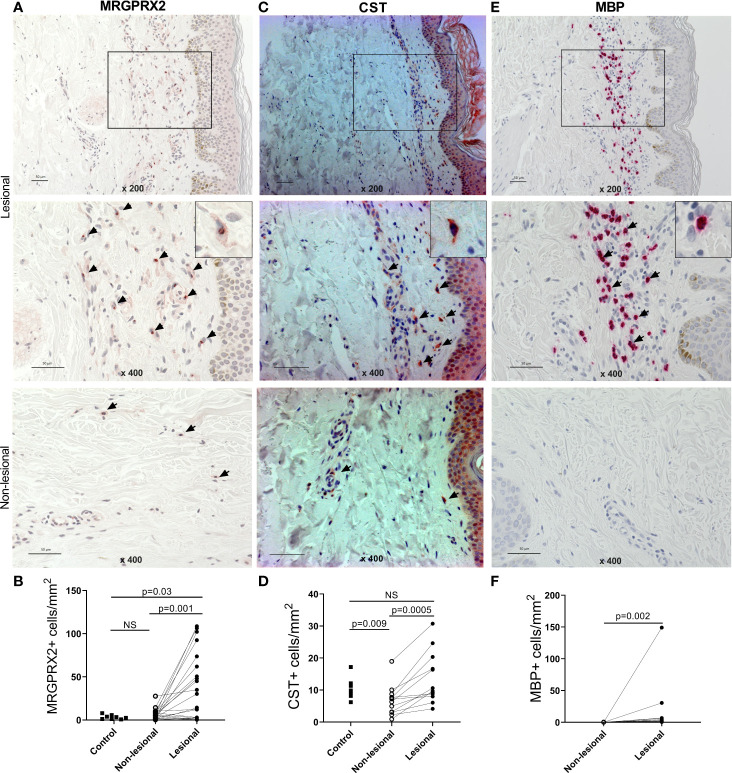
The number of MRGPRX2+, CST+, and MBP+ cells is increased in the lesions of patients with indolent systemic mastocytosis. Immunohistochemical staining of **(A)** MRGPRX2, **(C)** CST, and **(E)** MBP in the lesional and non-lesional skin of patients with ISM #6, #10, and #13, respectively. ×200 magnification of upper picture; ×400 magnification of all other pictures (expressing cells are shown by arrows). Bar = 50 µm. Number of **(B)** MRGPRX2+ cells, **(D)** CST+ cells, and **(F)** MBP+ cells in lesional and non-lesional skin of patients with ISM and skin of healthy controls. p < 0.05 was considered significant. The number of patients and controls as well as means/medians are summarized in the **Table 2**. CST, cortistatin; ISM, indolent systemic mastocytosis; MBP, major basic protein; NS, non-significant.

The number of MRGPRX2-expressing (MRGPRX2+) cells was significantly higher in lesional skin (median [IQR]: 22.3 [2.5–64.8] cells/mm^2^) as compared to non-lesional skin (5.2 [1.5–9.0] cells/mm^2^, p = 0.001) and skin of HCs (2.9 [1.2–5.2] cells/mm^2^, p = 0.03, **Table 2**, **Figures 1A, B**; **Supplementary Figure 2A**). Most cells showed intracellular and/or paranuclear expression. The membrane expression of MRGPRX2 was rarely detected and more frequently observed in patients with ISM compared to HCs.

**Table 2:** MRGPRX2 and its agonists in the skin of ISM patients and healthy controls.

Parameter	ISM		HCs	P value
	**Lesional (L)**	**Non-lesional (NL)**	2.9 (1.2-5.2) *n=8*	0.001 (L vs. HCs) 0.03 (L vs. NL)
**MRGPRX2+ cells/mm^2^**, median (IQR)	22.3 (2.5–64.8) *n = 22*	5.2 (1.5–9.0) *n = 21*		
**MRGPRX2-mRNA+ MCs/mm^2^**, median (IQR)	12.4 (3.6–21.5) *n = 8*	4.3 (0.3–9.8) *n = 8*	–	0.017
**MRGPRX2-mRNA+ MCs in all MCs, %**, median (IQR)	15.3 (7.7–23.1) *n = 8*	13.3 (0.6–32.8) *n = 8*	–	NS
**MRGPRX2-mRNA+ MCs in all MRGPRX2-mRNA+ cells, %**, mean ± SD (range)	32.0 ± 23.8 (2.9–79.1) *n = 8*	19.2 ± 22.5 (0.0–57.0) *n = 8*	–	0.05
**CST+ cells/mm^2^** Mean ± SD (range)	13.8 ± 8.1 (4.1–30.8) *n = 12*	6.7 ± 4.8 (0.9–19.0) *n = 12*	10.7 ± 3.0 (6.2–17.2) *n = 10*	0.0005 (L vs. NL) 0.009 (NL vs. HCs)
**MBP+ cells/mm^2^** median (IQR)	0.7 (0.0–3.6) *n = 22*	0.0 (0.0–0.0) *n = 21*	–	0.002

CST, cortistatin; ISM, indolent systemic mastocytosis; HCs, healthy controls; L, lesional skin; NL, non-lesional skin; NS, non-significant; –, no information.

The mean number of CST-expressing (CST+) cells was significantly higher in lesional skin than non-lesional skin (mean ± SD: 13.8 ± 8.1 vs. 6.7 ± 4.8 cells/mm^2^, p = 0.0005). CST+ cells were also more prominent in lesional skin than in skin of HCs (10.7 ± 3.0 cells/mm^2^); however, statistical significance was not reached (**Figures 1C, D**; **Supplementary Figure 2B**).

The number of MBP-expressing (MBP+) cells was significantly higher in lesional skin as compared to non-lesional skin (median [IQR]: 0.7 [0.0–3.6] vs. 0.0 [0.0–0.0] cells/mm^2^, p = 0.002) (**Figures 1E, F**).

Mean/median, IQR, and range values are summarized in **Table 2**.

### The Number of Mast Cells and MRGPRX2-mRNA+ Mast Cells Is Increased in the Skin of Patients With Indolent Systemic Mastocytosis

Next, we performed IF staining and *in situ* hybridization in combination with IF to identify if and how many MCs are MRGPRX2+ and MRGPRX2-mRNA+. Moreover, we compared the percentage of MRGPRX2-mRNA+ MCs in lesional and non-lesional skin.

**Figure 2:**
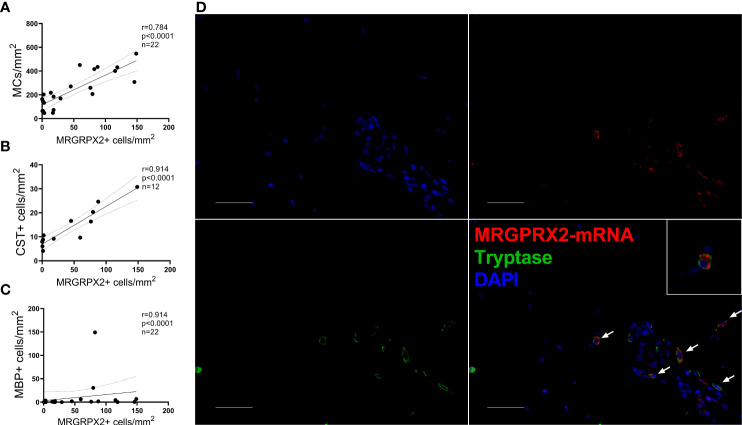
Mast cells express MRGPRX2, and their numbers correlate with numbers of MRGPRX2+ cells in ISM patients. In lesional skin, the number of MRGPRX2+ cells correlates with the number of **(A)** MCs, **(B)** CST+, and **(C)** MBP+ cells. The Spearman correlation coefficient was applied to assess correlations. **(D)** Immunofluorescence staining of lesional skin of a patient with ISM #12 with anti-tryptase (green), anti-MRGPRX2 (red), and DAPI (blue). White arrows indicate some of tryptase-MRGPRX2 double-positive cells. ×400 magnification. Bar = 50 µm. CST, cortistatin; DAPI, 49-6-diamidino-2-phenylindole dihydrochloride; MBP, major basic protein; MC, mast cell.

The number of MRGPRX2+ cells strongly correlated with the number of MCs and CST+ and MPB+ cells (**Figures 2A–C**). Double staining for MRGPRX2 and MC tryptase strongly suggests for a colocalization of MRGPRX2 and MCs (**Figure 2D**).

**Figure 3:**
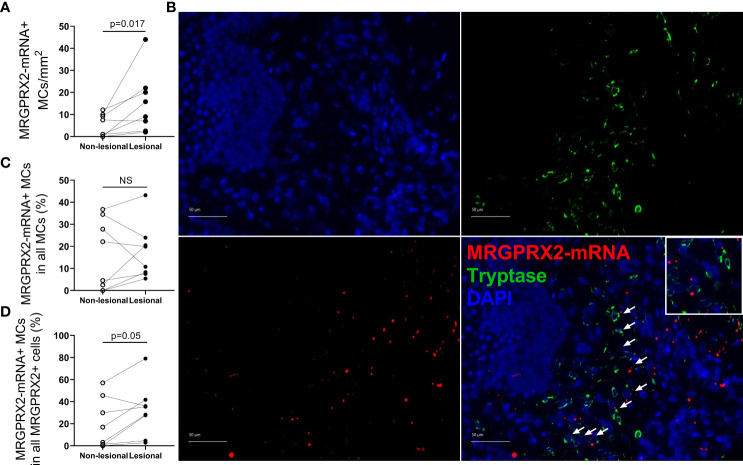
The number of MRGPRX2-mRNA-expressing mast cells is increased in lesional skin of patients with indolent systemic mastocytosis. **(A)** Number of MRGPRX2-mRNA+ MCs in lesional and non-lesional skin of ISM patients. **(B)** *In situ* hybridization (MRGPRX2-mRNA) (red) and immunofluorescence (tryptase) (green) double staining in lesional skin of a patient with ISM #12 (MRGPRX2-mRNA+MCs are shown by white arrows). ×400 magnification. Bar = 50 µm. **(C)** The percentage of MRGPRX2-mRNA+ MCs in all MCs in lesional and non-lesional skin of ISM patients. **(D)** The percentage of MRGPRX2-mRNA+ MCs in all MRGPRX-mRNA+ cells in lesional and non-lesional skin of ISM patients. ISM, indolent systemic mastocytosis; MC, mast cell.

The number of MRGPRX2-mRNA+ MCs in ISM patients was markedly higher in lesional than non-lesional skin (median [IQR]: 12.4 [3.6–21.5] vs. 4.3 [0.3–9.8] cells/mm^2^, p = 0.017) (**Figures 3A, B**), whereas the overall median percentage of MRGPRX2-mRNA+ MCs in all of MCs was rather low (<16%) and did not differ between lesional and non-lesional skin (**Table 2**; **Figure 3C**). The mean percentage of MRGPRX2-mRNA+ MCs in all of MRGPRX2-mRNA+ cells in ISM patients was significantly higher in lesional skin as compared to non-lesional skin, i.e., 32.0 ± 23.8% and 19.2 ± 22.5%, respectively (p = 0.05) (**Table 2**; **Figure 3D**).

### Increased Numbers of MRGPRX2+ Cells, MRGPRX2-mRNA+ MCs, and CST+ and MBP+ Cells are Not Associated With Clinical and Laboratory Features of ISM

Finally, we aimed to assess the association of MRGPRX2+ cells, MRGPRX2-mRNA+ MCs, and CST+ and MBP+ cells in the skin with clinical and laboratory characteristics of ISM patients.

**Figure 4:**
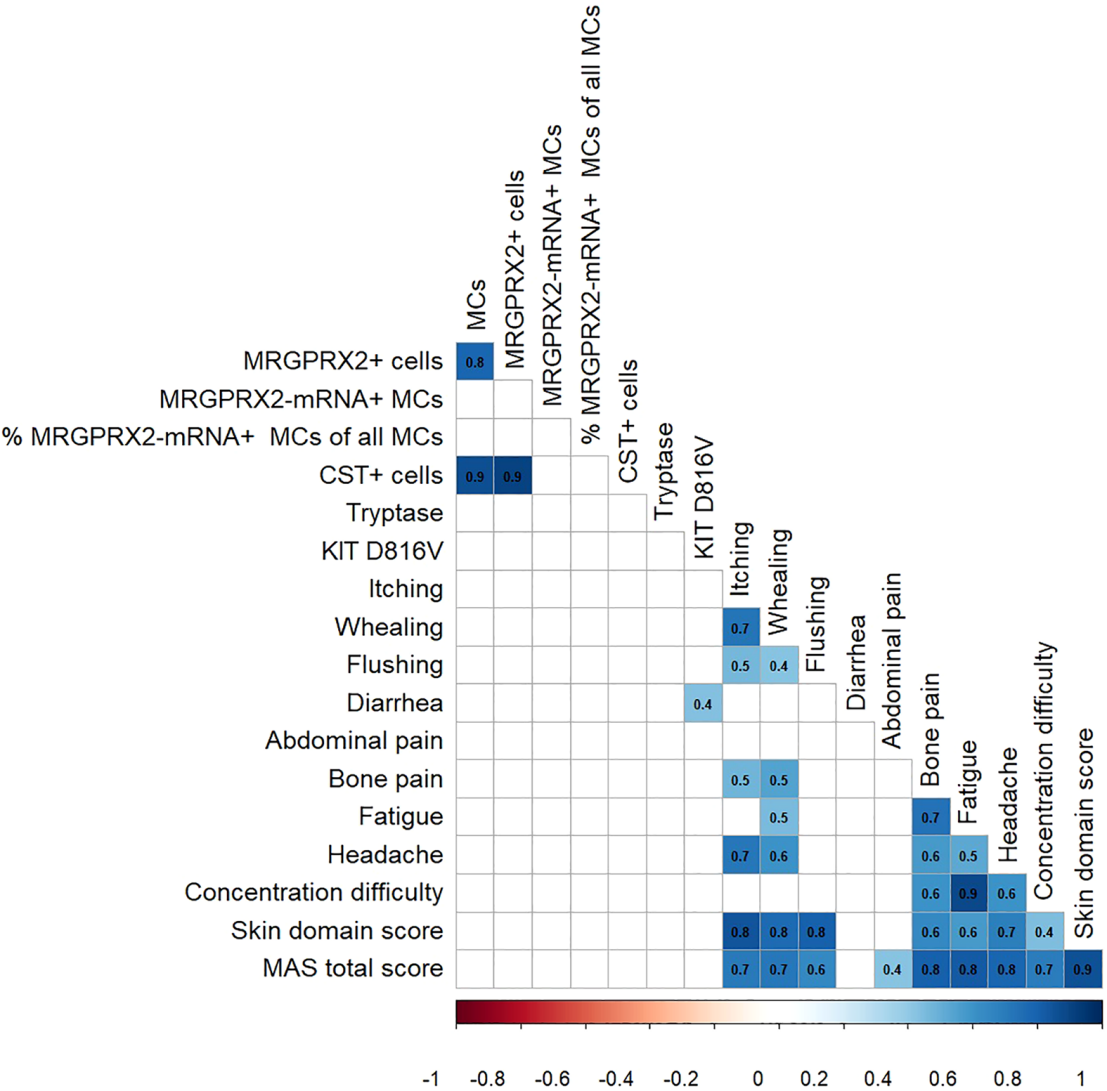
Correlation of MRGPRX2 and its agonists in the skin with the clinical and laboratory parameters in patients with ISM.The color shades represent the value of the Spearman correlation coefficient (r) and the relationship (red and blue are negative and positive r values, respectively). Only statistically significant correlations are shown (p < 0.05). CST, cortistatin; MAS, mastocytosis activity score; MC, mast cell.

There was no link between the numbers of MRGPRX2+ cells, MRGPRX2-mRNA+ MCs, and CST+ and MBP+ cells in lesional skin of ISM patients and disease clinical characteristics, i.e., evidence of anaphylaxis and/or osteopenia/osteoporosis, severity of mastocytosis in the skin (MIS score), and symptom burden as assessed by MAS (**Figure 4**; **Supplementary Figure 3**). Neither the number of MRGPRX2+ cells nor the number or percentage of MRGPRX2-mRNA+ MCs in the skin of ISM patients correlated with any laboratory parameter, including bST, *KIT* D816V allele burden, and alkaline phosphatase levels (**Figure 4**).

## Discussion

In this study, we intended to identify whether the presence of MRGPRX2 or its agonists in the skin is associated with certain symptoms, particularly itch and flushing, in patient with ISM. We found that skin lesions of ISM patient hosts markedly increased numbers of MRGPRX2+, CST+, and MPB+ cells and MRGPRX2-mRNA+ MCs, although this increase was not associated with severity of signs and symptoms in ISM patients.

MCs are known to express MRGPRX2, and MC accumulation is a typical finding in mastocytosis in the skin (7). Indeed, overexpression of MRGPRX2 and/or increased numbers of MRGPRX2+ MCs have been described in the lesions of patients with MC-driven disorders, e.g., cutaneous mastocytosis (13), chronic spontaneous urticaria (CSU) (15), and chronic prurigo (23), and correlated with disease severity in the latter. In line with this, the number of MRGPRX2+ cells was increased in our study and correlated with MC numbers as well as numbers of MBP+ cells. MBP, stored in eosinophil granules, has been described to activate human skin MCs *via* MRGPRX2 (15). Eosinophils, *via* cross talk with MCs, are known to contribute to various inflammatory and neoplastic disorders (24). They were identified in lesional skin of most patients with mastocytosis, varying from minimal to severe eosinophil infiltration, with the latter being present after initiation of a wheal-and-flare reaction (Darier’s sign) (25). A study by Wedi et al. showed that blood eosinophils express MRGPRX2 but to a lesser extent than MCs (26). We observed elevated numbers of MBP+ cells in lesional skin of ISM patients, although the overall number in the skin was rather low. However, MBP levels exhibited no correlation with signs in ISM patients and were not linked to symptom severity. Whether other cells, e.g., eosinophils or neurons, express MRGPRX2 in the skin of ISM patients should be investigated in further studies. In our study, the number of CST+ cells was also higher in lesional skin of patients with ISM and correlated with the extent of MC accumulation. CST is one of the most potent MRGRPX2 agonists and has been reported to be expressed and released by MCs upon activation (16, 23). This neuropeptide is thought to have both pro- and anti-inflammatory activities (27) and was shown to be upregulated in the skin of patients with chronic prurigo (23), but downregulated in psoriasis (28). In chronic prurigo, proinflammatory properties of CST might be related to autocrine activation of MCs *via* MRGPRX2 (23). In our study, increased numbers of CST+ cells were not associated with ISM symptom severity, including skin symptoms. However, the exact role of CST in the pathophysiology of ISM and its clinical relevance should be assessed in further studies.

Despite increased numbers of MRGPRX2+ cells, there was no association of any MRGPRX2-related parameter with clinical or laboratory features of ISM in our cohort of patients. There was no difference in MRGPRX2+ MC burden in the skin in patients with and without anaphylaxis, which is in line with recently published data (22). Whether anaphylaxis in ISM patients is linked to IgE-dependent or MRGPRX2-dependent MC activation or both mechanisms needs to be investigated in further studies. FcεRI- and MRGPRX2-evoked MC degranulation has been recently shown to be fully additive (29).

Finally, we assessed whether MRGPRX2 expression differs between wild-type MCs and MCs with *KIT* D816V mutation using a recently developed human-induced pluripotent stem cell-derived mast cell model (hiPSC-MCs) (30). The surface expression of MRGPRX2 on hiPSC-MCs carrying the *KIT* D816V mutation (hiPSC-MCs*^KIT^* ^D816V^) as assessed by flow cytometry was comparable to the respective wild-type (hiPSC-MCs^WT^) (data not shown). Thus, we can speculate that the *KIT* D816V mutation does not affect the level of MRGPRX2 surface expression on MCs.

Our study is limited by inclusion of only symptomatic patients and a single-center retrospective design. In most cases, a functional mechanism for the elicitation of symptoms by MRGPRX2 agonists could not be evaluated in detail. The role and relevance of MRGPRX2 and its agonists for the elicitation of anaphylaxis and intolerance reactions to different triggers, e.g., drugs, in ISM should be addressed in future studies.

In conclusion, skin lesions of patients with ISM showed high numbers of MRGPRX2-expressing cells, although the *KIT* D816V mutation, at least *in vitro*, seems not to affect MRGPRX2 expression by MCs. The expression of MRGPRX2 and its agonists in the skin exhibits no association with signs and symptoms of ISM. Whether other factors, such as a combination of different MRGPRX2 agonists, synergy with the FcεRI-dependent pathway, or MRGPRX2 genetic alterations, are being involved in MC activation in patients with ISM is yet unknown. To answer these questions, further studies should be performed, e.g., functional assays and *in vivo* tests with MRPGRX2 agonists.

## Data Availability Statement

The original contributions presented in the study are included in the article/**Supplementary Material**. Further inquiries can be directed to the corresponding author.

## Ethics Statement

The studies involving human participants were reviewed and approved by the Ethics Committee of Charité – Universitätsmedizin Berlin EA1/407/16. The patients/participants provided their written informed consent to participate in this study.

## Author Contributions

PP, FS, and PK designed the study, interpreted the data, and prepared the manuscript. PP, PK, SM-R, YL, TA, HB, QJ, JH, and MH (8th Author) performed experiments and/or analyzed the data. The study was supervised by PK and FS. All coauthors critically revised and provided substantial input to the manuscript. All authors contributed to the article and approved the submitted version.

## Funding

This study was in part financially supported by Allakos Inc. The funder was not involved in the study design, collection, analysis, interpretation of data, the writing of this article, or decision to submit it for publication.

## Conflict of Interest

HB received honoraria (advisory board, speaker) from AbbVie, Novartis, and Sanofi-Aventis, outside of submitted work. JS has no relevant conflict of interest in relation to this work. Outside of it, JS is or recently was advisor for Boehringer Ingelheim. BY is employed by Allakos Inc. MM has no relevant conflict of interest in relation to this work. Outside of it, MM is or recently was a speaker and/or advisor or received institutional research funding from Astria, Allakos, Alnylam, Amgen, Aralez, ArgenX, AstraZeneca, BioCryst, Blueprint, Celldex, Centogene, CSL Behring, Dyax, FAES, Genentech, GIInnovation, GSK, Innate Pharma, Kalvista, Kyowa Kirin, Leo Pharma, Lilly, Menarini, Moxie, Novartis, Pfizer, Pharming, Pharvaris, Roche, Sanofi/Regeneron, Shire/Takeda, Third Harmonic Bio, UCB, and Uriach. FS is or recently was a speaker and/or advisor for and/or has received research funding from Allakos, Blueprint, Celldex, CogentBio, Genentech, Novartis, Moxie, Sanofi/Regeneron, and Uriach. PK has no relevant conflict of interest in relation to this work. Outside of it, PK is or recently was a speaker and/or advisor for Novartis and Roche.

The remaining authors declare that the research was conducted in the absence of any commercial or financial relationships that could be construed as a potential conflict of interest.

## Publisher’s Note

All claims expressed in this article are solely those of the authors and do not necessarily represent those of their affiliated organizations, or those of the publisher, the editors and the reviewers. Any product that may be evaluated in this article, or claim that may be made by its manufacturer, is not guaranteed or endorsed by the publisher.

## Abbreviations

ASO-qPCR, allele-specific quantitative polymerase chain reaction; bST, baseline serum tryptase; CST, cortistatin; ECP, eosinophil cationic protein; IF, immunofluorescence; IHC, immunohistochemistry; ISH, *iIn situ* hybridization; ISM, indolent systemic mastocytosis; MAS, mastocytosis activity score; MBP, major basic protein; MC, mast cell; MIS, mastocytosis in the skin; MRGPRX2, Mas-related G-protein coupled receptor X2; PB, peripheral blood; SP, substance P; WHO, World Health Organization.
