# Supplementary material for: The Number of MRGPRX2-Expressing Cells Is Increased in Skin Lesions of Patients With Indolent Systemic Mastocytosis, But Is Not Linked to Symptom Severity
Source: Front Immunol. 2022 Jul 26;13:930945. doi: 10.3389/fimmu.2022.930945 (PMC9361751; doi:10.3389/fimmu.2022.930945)
Supplement: Supplementary file 1 [file DataSheet_1.docx]

**Supplementary Figure 1. Mastocytosis in the skin score (MIS score).**

Images show representative examples for different grades of mastocytosis in the skin. Grading was assessed in the most affected hand-sized area by analyzing the number and color intensity of lesions.

Grade 0: no skin involvement

Grade 1: limited skin involvement (<10% area involved, light red/brown color)

Grade 2: mild skin involvement (10-30% area involved, light red/brown color)

Grade 3: moderate skin involvement (20-50% area involved, red/brown color)

Grade 4: severe skin involvement (50-70% area involved, red/brown color)

Grade 5: very severe skin involvement (>70% area involved, almost confluent lesions)

**Supplementary Figure 2**. **MRGPRX2- and CST-expressing cells are limited in healthy skin.**

Immunohistochemical staining of **(A)** MRGPRX2 and **(B)** CST in the skin of HC #3 and #1, respectively (expressing cells are shown by arrows). x400 magnification. Bar = 50 µm

**Supplementary Figure 3.**  **The MRGPRX2-agonists and MRGPRX2 in the skin of ISM patients with and without anaphylaxis and with different disease burden and skin involvement.**

(**A**) MRGPRX2+ cells/mm^2^, (**B**) MRGPRX2-mRNA+ MCs/mm^2^, (**C**) CST+ cells/mm^2^ and (**D**) MBP+ cells/mm^2^ in lesional skin of ISM patients with and without anaphylaxis. (**E**) MRGPRX2+ cells/mm^2^, (**F**) MRGPRX2-mRNA+ MCs/mm^2^, (**G**) CST+ cells/mm^2^ and (**H**) MBP+ cells/mm^2^ in lesional skin of ISM patients with mild/moderate and severe skin symptoms (itching, whealing and flushing). (**I**) MRGPRX2+ cells/mm^2^, (**J**) MRGPRX2-mRNA+ MCs/mm^2^, (**K**) CST+ cells/mm^2^ and (**L**) MBP+ cells/mm^2^ in lesional skin of ISM patients with mild, moderate and severe disease burden, based on total MAS score. (**M**) MRGPRX2+ cells/mm^2^, (**N**) MRGPRX2-mRNA+ MCs/mm^2^, (**O**) CST+ cells/mm^2^ and (**P**) MBP+ cells/mm^2^ in lesional skin of ISM patients with limited, mild, moderate, severe and very severe MIS, based on total MIS score.

Differences between two groups were calculated with the Mann-Whitney U-test (not normally distributed data) and t-test (normally distributed data). Differences between more than two groups were calculated with the Kruskall-Wallis test with Bonferroni correction. The differences between groups were not significant and are not shown on the graph.

MAS: Mastocytosis Activity Score; MIS score: mastocytosis in the skin score
