# Supplementary figures and images for: The Number of MRGPRX2-Expressing Cells Is Increased in Skin Lesions of Patients With Indolent Systemic Mastocytosis, But Is Not Linked to Symptom Severity
Source: Front Immunol. 2022 Jul 26;13:930945. doi: 10.3389/fimmu.2022.930945 (PMC9361751; doi:10.3389/fimmu.2022.930945)

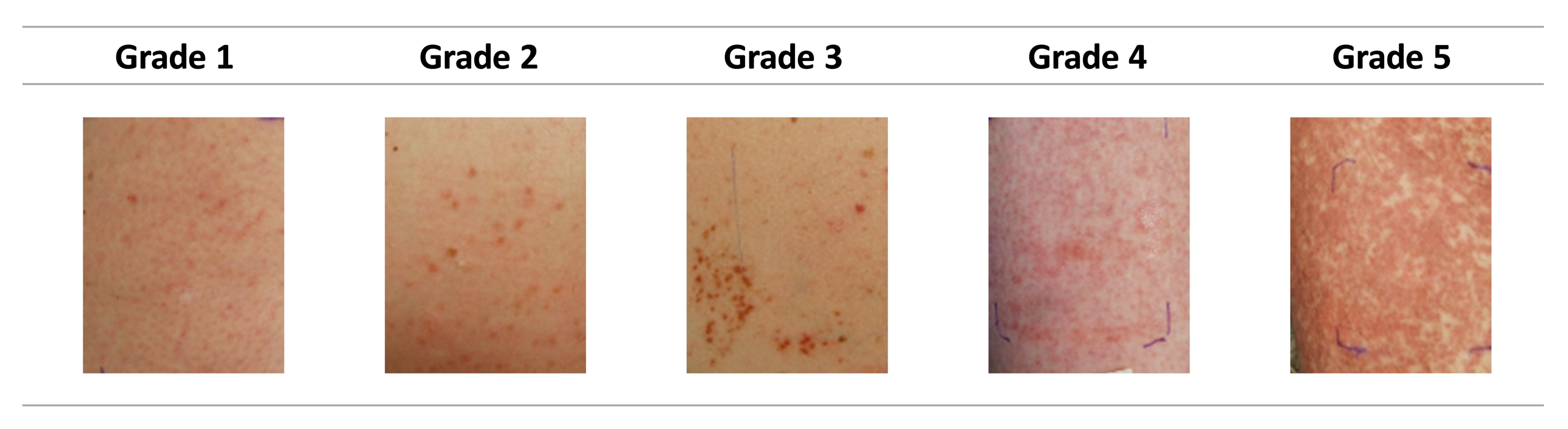

Supplement: Supplementary file 2 [file Image_1.jpeg]

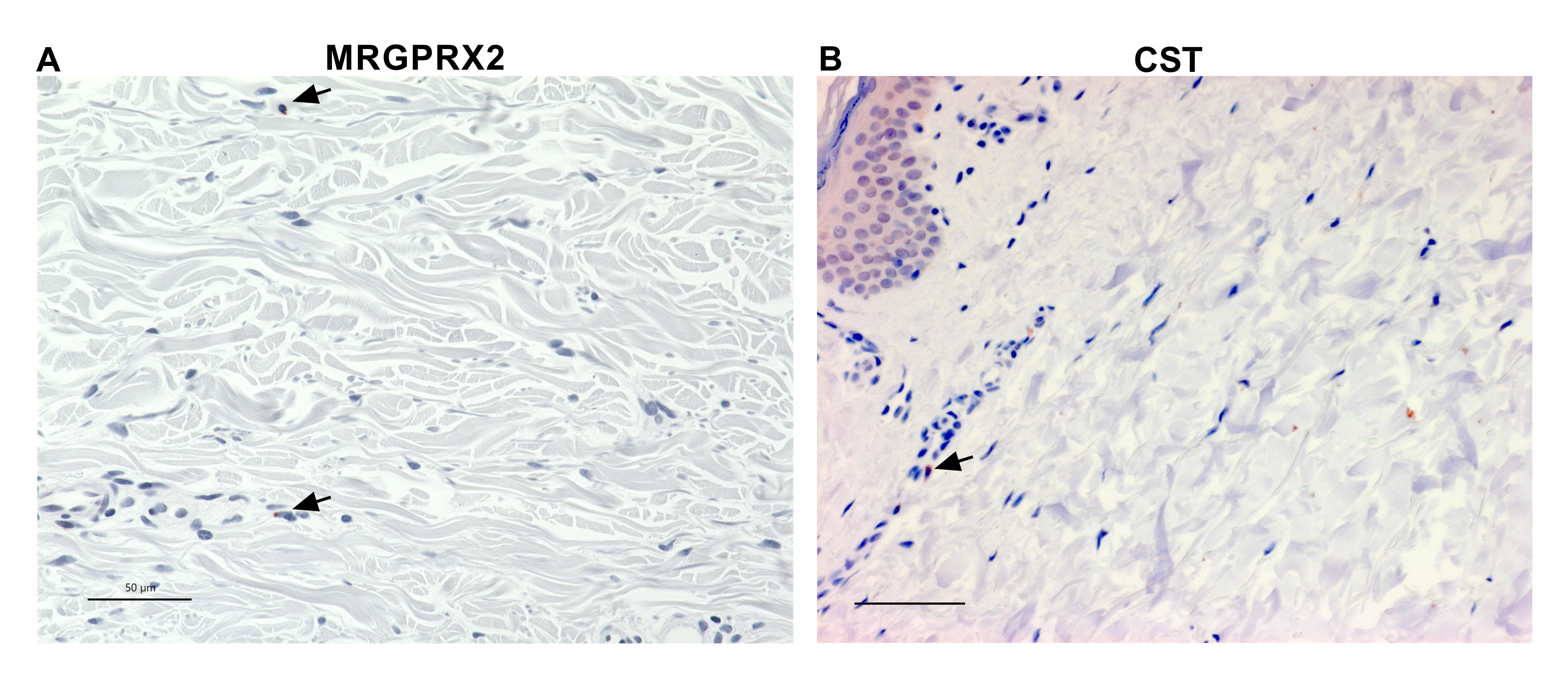

Supplement: Supplementary file 3 [file Image_2.jpeg]
