# Supplementary material for: The Number of MRGPRX2-Expressing Cells Is Increased in Skin Lesions of Patients With Indolent Systemic Mastocytosis, But Is Not Linked to Symptom Severity
Source: Front Immunol. 2022 Jul 26;13:930945. doi: 10.3389/fimmu.2022.930945 (PMC9361751; doi:10.3389/fimmu.2022.930945)
Supplement: Supplementary file 5 [file Table_1.docx]

**Supplementary Table 1. Characteristics of ISM patients**

| **ID** | **Age, y** | **Sex** | **Anaphylaxis** | **Osteoporosis/ osteopenia** | **MIS score** | **MAS** | ***KIT* D816V burden, %** | **bST, ng/ml** |
| --- | --- | --- | --- | --- | --- | --- | --- | --- |
| 1 | 33 | M | – | – | 2 | 22 | 0.37 | 32.8 |
| 2 | 60 | F | no | no | 5 | 17 | 2.10 | 162.0 |
| 3 | 54 | F | yes | yes | 1 | 23 | 0.48 | 82.5 |
| 4 | 63 | F | no | – | 2 | 32 | neg. | 16.4 |
| 5 | 61 | F | yes | yes | 3 | 32 | 2.00 | 27.0 |
| 6 | 58 | F | yes | yes | 4 | 29 | 1.00 | 21.4 |
| 7 | 51 | F | yes | yes | 2 | 15 | 3.00 | 16.2 |
| 8 | 56 | M | no | yes | 3 | 52 | 0.31 | 61.1 |
| 9 | 47 | F | no | yes | 2 | 10 | 0.10 | 28.4 |
| 10 | 39 | M | no | no | 3 | 15 | 0.30 | 109.0 |
| 11 | 44 | F | yes | no | 2 | 48 | 1.10 | 52.1 |
| 12 | 47 | M | no | yes | 5 | 48 | 6.90 | 168.0 |
| 13 | 64 | F | no | yes | 3 | 38 | 1.70 | 23.0 |
| 14 | 58 | F | no | yes | 1 | 50 | 0.39 | 6.4 |
| 15 | 50 | F | yes | yes | 3 | 40 | 17.00 | 111.0 |
| 16 | 42 | F | no | no | 4 | 44 | 27.00 | 119.0 |
| 17 | 65 | M | no | no | 1 | 16 | 0.90 | 172.0 |
| 18 | 42 | F | no | no | 5 | 28 | 53.00 | 96.6 |
| 19 | 40 | F | yes | yes | 2 | 45 | 0.23 | 29.1 |
| 20 | 40 | F | no | yes | 2 | 54 | 0.48 | 22.2 |
| 21 | 53 | F | yes | yes | 1 | 24 | 1.90 | 16.9 |
| 22 | 46 | M | yes | yes | 2 | 9 | 0.13 | 19.9 |

bST: baseline serum tryptase; – no information; F: female; M: male; MAS: mastocytosis activity score; MIS: mastocytosis in the skin
