# Supplementary material for: The Number of MRGPRX2-Expressing Cells Is Increased in Skin Lesions of Patients With Indolent Systemic Mastocytosis, But Is Not Linked to Symptom Severity
Source: Front Immunol. 2022 Jul 26;13:930945. doi: 10.3389/fimmu.2022.930945 (PMC9361751; doi:10.3389/fimmu.2022.930945)
Supplement: Supplementary file 6 [file Table_2.docx]

**Supplementary Table 2. Characteristics of healthy controls.**

| **ID** | **Age, y** | **Sex** | **bST, ng/ml** |
| --- | --- | --- | --- |
| 1 | 41 | M | 4,21 |
| 2 | 52 | F | 6,30 |
| 3 | 37 | F | 5,28 |
| 4 | 23 | M | 2,89 |
| 5 | 55 | M | 5,73 |
| 6 | 54 | F | 4,69 |
| 7 | 68 | M | 7,21 |
| 8 | 62 | M | 4,10 |
| 9 | 57 | M | 6,37 |
| 10 | 67 | F | 5,10 |

bST: baseline serum tryptase; – no information; F: female; M: male
